# Discovery and validation of gene classifiers for endocrine-disrupting chemicals in zebrafish (*danio rerio*)

**DOI:** 10.1186/1471-2164-13-358

**Published:** 2012-08-01

**Authors:** Rong-Lin Wang, David Bencic, Adam Biales, Robert Flick, Jim Lazorchak, Daniel Villeneuve, Gerald T Ankley

**Affiliations:** 1 USEPA, Ecological Exposure Research Division, National Exposure Research Laboratory, 26 W Martin Luther King Dr, Cincinnati, OH, 45268, USA; 2 USEPA, Mid-Continent Ecology Division, National Health and Environmental Effects Research Laboratory, 6201 Congdon Boulevard, Duluth, MN, 55804, USA

**Keywords:** Gene classifiers, Endocrine-disrupting chemicals, Transcriptomics, Mechanism of action, Zebrafish

## Abstract

**Background:**

Development and application of transcriptomics-based gene classifiers for ecotoxicological applications lag far behind those of biomedical sciences. Many such classifiers discovered thus far lack vigorous statistical and experimental validations. A combination of genetic algorithm/support vector machines and genetic algorithm/K nearest neighbors was used in this study to search for classifiers of endocrine-disrupting chemicals (EDCs) in zebrafish. Searches were conducted on both tissue-specific and tissue-combined datasets, either across the entire transcriptome or within individual transcription factor (TF) networks previously linked to EDC effects. Candidate classifiers were evaluated by gene set enrichment analysis (GSEA) on both the original training data and a dedicated validation dataset.

**Results:**

Multi-tissue dataset yielded no classifiers. Among the 19 chemical-tissue conditions evaluated, the transcriptome-wide searches yielded classifiers for six of them, each having approximately 20 to 30 gene features unique to a condition. Searches within individual TF networks produced classifiers for 15 chemical-tissue conditions, each containing 100 or fewer top-ranked gene features pooled from those of multiple TF networks and also unique to each condition. For the training dataset, 10 out of 11 classifiers successfully identified the gene expression profiles (GEPs) of their targeted chemical-tissue conditions by GSEA. For the validation dataset, classifiers for prochloraz-ovary and flutamide-ovary also correctly identified the GEPs of corresponding conditions while no classifier could predict the GEP from prochloraz-brain.

**Conclusions:**

The discrepancies in the performance of these classifiers were attributed in part to varying data complexity among the conditions, as measured to some degree by Fisher’s discriminant ratio statistic. This variation in data complexity could likely be compensated by adjusting sample size for individual chemical-tissue conditions, thus suggesting a need for a preliminary survey of transcriptomic responses before launching a full scale classifier discovery effort. Classifier discovery based on individual TF networks could yield more mechanistically-oriented biomarkers. GSEA proved to be a flexible and effective tool for application of gene classifiers but a similar and more refined algorithm, connectivity mapping, should also be explored. The distribution characteristics of classifiers across tissues, chemicals, and TF networks suggested a differential biological impact among the EDCs on zebrafish transcriptome involving some basic cellular functions.

## Background

Microarray-based high throughput transcriptomic analysis profiles transitory changes in gene expression of an organism in response to perturbations such as a disease state or exposure to chemical or non-chemical stressors. A selected set of characteristic gene signatures could serve as a diagnostic or prognostic tool for samples of interest with regard to a possible perturbation. Since the development of microarray technology in the 1990’s, there has been a steadily increasing interest in developing these gene signatures for various clinical and environmental applications [1-4]. As a result, several gene classifiers^a^ were approved for use in human clinical diagnostics, and many more yet to be validated candidates have been identified for ecotoxicological purposes.

To date, most transcriptomics-based gene classifier studies have been conducted in the area of human clinical diagnosis and prognosis, often with mixed results. However, there have been several successful applications recently. For example, four subtypes of breast cancer were differentiated by expression profiling [5], and a 70-gene prognostic classifier was successfully developed and approved by the US Food and Drug Administration to characterize breast cancer patient risks [6]. The Pathwork Tissue of Origin Test, a microarray-based gene expression test involving 1500 genes, was also shown to be capable of determining the tissue of origin of poorly or undifferentiated cancers, thus facilitating their diagnosis [7]. Perhaps the most encouraging success so far is the concept of connectivity mapping (Cmap), where chemical compounds with similar/dissimilar mechanisms of action (MOAs) and disease states can be connected by assessing a group of their gene signatures relative to a reference collection of gene expression profiles (GEPs) [8]. Despite these notable successes, there remain issues that need to be resolved before a widespread acceptance and application of gene classifier approaches become reality, perhaps particularly so in ecotoxicology. For example, the stability/reliability of gene classifiers across independent studies is a common concern. Often, classifiers developed for the same phenotype from different studies have little overlap and perform rather poorly on independent datasets [9]. And many proposed ecotoxicological gene classifiers are simply differentially expressed genes lacking vigorous statistical and experimental validations.

The predictive performance of a gene classifier is dependent on several technical and biological factors, including quality of data acquisition, choice of classification algorithm, and the complexity of the dataset under investigation. This data complexity refers to the extent to which a dataset is intrinsically structured such that classes of treated and control samples can be separated into distinct groups as defined by gene features (i.e. a geometric boundary can be established computationally between classes in a multi-dimensional space). When data quality and classification algorithms are controlled, for example within the same study, the difference in complexity among chemical-tissue conditions underlying gene classifiers may become critical to their respective performance [10]. This relationship between data complexity and the performance of a gene classifier was addressed in several recent studies [11-14]. A number of measures such as Fisher’s discriminant ratio (F-ratio), overlap between the distributions of two classes, and feature efficiency have been proposed to capture this complexity [10]. Studies comparing cancer biomarkers suggested that their performance is closely linked to data complexity and sample size [15]. When this function is not optimized, there is an increased risk of model overfitting, where stochastic noise in training data gets incorporated and results in poor classifiers. An assessment of classifier performance as a function of data complexity in an ecotoxicological context, therefore, could generate significant empirical insights concerning issues such as sample size required for discovery of a particular type of gene classifier in an optimized experimental design.

Application of microarray technology, in general, and development of microarray-based gene classifiers, in particular, for ecotoxicological applications lags far behind those in human biomedical sciences. Despite numerous candidate molecular indicators discovered in recent years, few are field-ready for exposure and risk assessment of environmental stressors. Several issues probably contribute to this relative lack of progress. First, there is a general lack of knowledge of the data complexity of individual chemical stressors. It is not clear what sample size is required for discovery of a gene classifier for any given chemical with a unique MOA. Much of the published literature on gene classifier discovery in ecotoxicology involved only a minimum number of biological replicates across treatment conditions due to the prohibitive cost of microarray technology, despite the desirability of a much larger number of replicates [15,16]. Second, very few studies have actually conducted independent validations on targeted candidate gene classifiers, a critical step neglected in the discovery process. Without vigorous validation, it is not at all clear how reliable putative gene classifiers are for chemical exposures. Finally, ecotoxicological application of gene classifiers is also complicated by a large number of uncontrolled physical, chemical, and biological variables in a field setting relative to human clinical applications.

The purpose of this study was to discover gene classifiers for a number of endocrine-disrupting chemicals (EDCs) with different MOAs, evaluate their performance, and gain insights on issues important to the discovery process in general, through vigorous computational search and statistical analyses with an experimental design and sampling strategy similar to those found in the current ecotoxicological literature. In addition, the summary statistics of classifiers by various search categories may also shed additional light on the biological impact of these EDCs. Specifically, our objectives were to: 1) search for EDC classifiers either across the entire zebrafish transcriptome or within individual transcription factor (TF) networks based on the microarray data from EDC-exposed fish samples; 2) generate additional microarray data dedicated to validation only and test the performance of selected candidates; 3) evaluate the correlation between the performance of classifiers and the data complexity of individual chemical-tissue conditions as measured by F-ratio; 4) and assess the relative effects of EDCs on zebrafish based on the summary statistics of classifiers across chemicals, tissue types, and TF networks.

## Methods

### Exposure, sampling, RNA extraction, and microarray profiling

As part of a larger computational toxicology project studying the impact of a number of EDCs on the hypothalamic-pituitary-gonadal (HPG) axis in fish, approximately 300 microarrays covering 58 treatment conditions for zebrafish were available for this investigation [17,18]. Many experiment details regarding the project, including the research goals, design, test chemicals, fish exposures, biological endpoints and sample handling (e.g., RNA extraction and hybridization) are available elsewhere [17,18]. Only a brief overview is provided below.

Zebrafish exposures were conducted using 10 chemicals with differing known/hypothesized MOAs within the HPG axis: 17α-ethynyl estradiol, fadrozole, 17β-trenbolone, fipronil, prochloraz, flutamide, muscimol, ketoconazole, trilostane, and vinclozolin [17]. Reproductively mature male and female zebrafish were exposed to a continuous flow of test chemical (two different, analytically-confirmed concentrations and a control), delivered in water (with no solvent), for 24, 48, or 96 h. At the end of each exposure period, fish were euthanized in a buffered solution of tricaine methanesulfonate (MS-222; Finquel, Argent, Redmond WA, USA) and tissues, including gonads, liver, and brains (with the pituitary gland and hypothalamus) were collected. Total RNA isolated from selected tissue samples was labeled and hybridized to microarrays by an Agilent certified contract laboratory (Cogenics, Morrisville, North Carolina 27560, USA). Expression profiling in zebrafish was achieved using Agilent two-color zebrafish microarrays (G2518A and G2519F, design 013223 and 015064, Agilent Technologies, Santa Clara, CA 95051, United States). Data from approximately 290 microarrays, representing 58 treatment conditions encompassing the 10 chemicals, three tissue types, and three exposure durations, in both male and female zebrafish were analyzed for candidate classifiers (Table 1). Additional unused 28 fish tissue samples of flutamide-ovary, prochloraz-ovary, and prochloraz-brain were set aside and subsequently profiled by microarrays for the validation of their corresponding classifiers. The entire microarray dataset is accessible through the National Center for Biotechnology Information Gene Expression Omnibus [19] with the accession number GSE38070.

**Table 1:** Sample size across chemical-tissue conditions for classifier search

**Chemical/tissue**	**Brain**	**Ovary**	**Testis**	**All tissues**
17α-Ethynyl Estradiol	10^b^	5	9	30
Fadrozole	15^c^	15	*NA*	30
Fipronil	10^c^	10	10	30
Flutamide	*NA*	10	15	25
Ketoconazole	10^c^	15	5	40
Muscimol	20^c^	5	*NA*	25
Prochloraz	10^d^	10	10	30
17β-Trenbolone	5	15	*NA*	25
Trilostane	*NA*	10/5	20 / 15	30/20
Vinclozolin	*NA*	10	15	25
Total	80	105	84	269/290

Except for trilostane-ovary and trilostane-testis, each entry represents equal number of independent replicates of both treated and control samples^a^. In trilostane where some of the same controls were paired to different treatment conditions, only unique ones were counted. Conditions with less than nine samples in either class were excluded from classifier search. “NA”, a condition not included in the study. The dataset of “all tissues” includes brain, ovary, testis, as well as a few additional samples from liver for selected conditions.

^a^for example, 17α-Ethynyl Estradiol-brain had 10 treated samples and 10 control samples; ^b^male only; ^c^male and female; ^d^female only.

### Gene classifier discovery and validation

**Figure 1:**
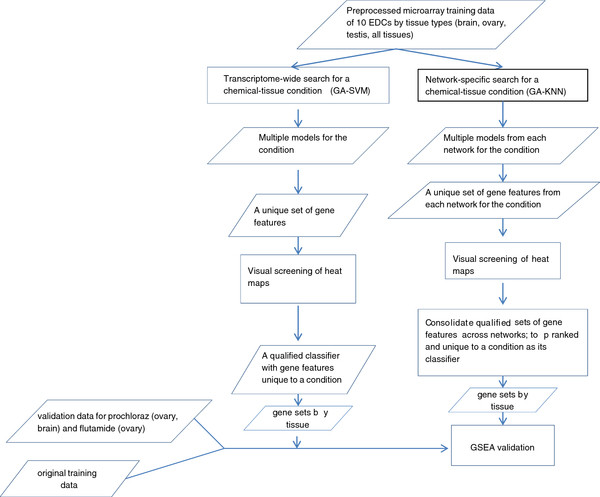
Flow chart for gene classifier discovery and validation.

Putative classifiers for various chemical-tissue conditions were identified through intensive computational searches with two different strategies (Figure 1, Additional file 1: Figure S1). While there are a great number of algorithms available for microarray-based gene classifier discovery, a choice was made to employ a combination of either GA-SVM (genetic algorithm-support vector machines) or GA-KNN (K-nearest neighbors) based on their previous evaluations as being the best and second best performer respectively [20]. GA is a computational approach modeled on biological evolution, incorporating concepts and processes such as genes, chromosomes, selection, recombination, mutation, and fitness [21]. It is a powerful tool for optimization, and thus well-suited for the task of reducing data dimensionality by selecting a small number of informative gene features among tens of thousands present on a microarray, a necessary first step for any classifier discovery algorithm. Genes selected and arranged into chromosomes then evolve under the principles of biological evolution employing either SVM or KNN as a fitness function. The fitness here is equivalent to the performance of a classifier. SVM can handle both linearly separable and non-separable microarray data [13,22]. The algorithm projects samples from a training dataset into high-dimensional space separated by a hyperplane [23], which is captured in a SVM model to be used subsequently for classifying unknown samples. KNN identifies a sample in question based on the class memberships of its nearest neighbors as determined by a distance measure in a multi-dimensional space defined by gene features [24].

The performance of gene classifiers was then evaluated by gene set enrichment analysis (GSEA), where a classifier containing a group of gene signatures associated with a treatment condition was determined for their non-random distribution on the rank-ordered gene lists from the GEPs of both training and validation data. If a classifier formatted as a gene set is enriched in these gene lists, their respective underlying chemical-tissue conditions must have similar MOAs, leading to the validation of the classifier. On the original training data, GSEA was conducted for the classifiers of all the conditions. For the dedicated validation data, only classifiers for several selected chemical-tissue conditions were assessed. The identities of these conditions were known but withheld during analysis.

### Microarray data preparation and characterization

To avoid issues resulting from complications of tissue-specific expression [25], gene classifiers were searched primarily within a tissue type. Datasets were prepared in this investigation separately from samples of ovary (105 microarrays and 10 chemicals), testis (84 microarrays and seven chemicals), and brain (80 microarrays and seven chemicals). As a comparison, an inclusive dataset was also generated containing all samples from above, plus several more from liver. Several dye-swaps among the 300 microarrays were excluded. Individual microarrays were processed as single channel intensities (Cy5, Cyanine 5; and Cy3, Cyanine 3) since, for these studies, the two channels contained unique biological samples [18] and the search for a binary classifier was more straightforward. Data preprocessing including filtering and normalization was conducted by dataset, identical to that described by Wang et al. [18]. The data complexity of a chemical- tissue condition was captured by F-ratio statistic, an effective measure of microarray data complexity [11]:

(1) $$\left(f_{i}\right)\text{max}={\left(\mu_{1}-\mu_{2}\right)}^{2}/(\sigma_{1}{}^{2}+\sigma_{2}{}^{2}),i=1\text{to}N$$

where μ_1_, μ_2_, σ_1_^2^, σ_2_^2^ are the means and variances of the two classes in the condition, and *f*_*i*_ the estimated value for gene feature *i*.

### Choice of software

Both GA-SVM and GA-KNN were implemented through the software R [26] package GALGO [27]. The algorithms were implemented in such a way that during a search, samples would be split randomly into a training group versus a test group a number of times. To ensure a minimum number of samples in both groups for the algorithm to function, each chemical-tissue condition must have at least nine microarrays (18 biological samples) in order to be included in the search for a gene classifier.

### General search strategies

Both the transcriptome-wide searches by GA-SVM and the network-specific searches by GA-KNN were applied to the three tissue-specific datasets and the all tissue combined dataset. While these datasets contained data for multiple chemical-tissue conditions, each search was always conducted on an individual condition within a dataset. Several considerations were taken into account in the design of search strategies with regard to datasets, search scope, and algorithms. To avoid the dominant impact of tissue type on GEPs, searches were primarily conducted within individual tissue types. However, to demonstrate tissue effect on classifier discovery, the all tissue combined dataset was also analyzed. For each chemical-tissue condition, the search scope was either across the entire zebrafish transcriptome or limited to previously reverse-engineered, individual TF networks [18]. In other words, the sampling space for GA consisted of all the expressed genes in zebrafish or those belonging to a particular TF network only. Given the established linkage between these TF networks and EDC effects in zebrafish, this network-specific search could potentially produce more mechanistically-based classifiers. GA-KNN was used for the network-specific searches because it is computationally less intensive than GA-SVM, and the overall computing load for these searches was far greater than that of the transcriptome-wide searches as a result of hundreds of TF networks over multiple chemical/tissue conditions involved. To further reduce computing demand, network-specific searches for the all tissue combined dataset were limited to three of its chemical-tissue conditions.

### Transcriptome-wide search

All gene features remaining in a given dataset (brain, ovary, testis, or all tissue combined) after data preprocessing were included in the search space for GA-SVM. The number of features was 13339 in brain, 12706 in ovary, 14148 in testis, and 12802 in the all tissues-combined dataset. Prior to searches by GA-SVM, a “cost” parameter necessary for a selected SVM kernel function had to be determined for individual datasets. An R script was developed for this purpose to conduct a grid search using the R package e1071, an interface to C++ library libsvm [28]. The GA-SVM search was configured by the GALGO setup function configBB. VarSel, and is summarized in Additional file 2: Table S1. A GA-SVM search was conducted independently for 29 different chemical-tissue conditions.

### Network-specific search

A TF network consists of a TF regulator and a number of target genes under its transcriptional regulation. A TF regulator is also referred as a hub TF. Previously, we identified 515 reverse-engineered TF networks significantly impacted by EDCs [18]. Network-specific searches by GA-KNN were applied to these 515 networks as well as the two additional groups of genes: the hub TFs of the 515 TF networks compiled into a separate group termed master regulators, and a list of genes coding for proteins known to be involved in regulation of the HPG axis [29]. For simplicity, the master regulators and HPG axis will also be counted as TF networks hereafter in the context of classifier search. Given the number of chemicals, multiple tissue types, and hundreds of TF networks, a separate R script wrapper had to be developed for GALGO in order to batch process a large number of searches. GA-KNN searching was also configured by the GALGO setup function configBB. VarSel (Additional file 2: Table S1). Searches for classifiers were conducted for each chemical-tissue condition by the 517 networks, which translated to over 10,000 independent searches spread out over multiple nodes of a computer cluster with the Linux operating system.

### Prioritizing candidate classifiers

The nature of GA-SVM and GA-KNN was such that there were typically multiple models generated in a search for a given gene classifier, with each model reaching the same pre-defined fitness goal and containing a number of gene features. Some of these gene features were often redundant among models. In the case of the transcriptome-wide search, the union of these models was first determined. The resultant unique set of gene features was then visually screened for class separation by its corresponding heat map for a particular chemical-tissue condition. Since network-specific searching involved multiple TF networks within a chemical-tissue condition, unique sets of gene features, each derived from multiple models within a network, had to be visually screened by their respective heat maps first, and if qualified, merged across TF networks. Those gene features that were redundant across multiple chemical-tissue conditions were then removed in order to reduce ambiguity in future field applications where multiple chemical mixtures may be present. Otherwise, a classifier with a subset of gene features responsive to multiple chemicals will become less condition-specific. The remaining gene features unique to a single chemical-tissue condition were then each ranked by the number of TF networks in which it was selected into a model. The top 100 were chosen as a classifier for each condition. For both transcriptome-wide and network specific searches, a heat map was generated by an R script developed in house based on a unique set of gene features and the gene expression data of a given chemical-tissue condition from which these features were identified. Each heat map contained two-way clustering of samples and genes. During its visual screening, a unique set of gene features would qualify as a classifier (transcriptome-wide search) or for further consolidation across TF networks (network specific search) only if there was a complete separation of treated and control samples into two distinct clusters. The phrase “qualified classifier” was used only in this context throughout this study.

### Validation

Qualified classifiers from all of the chemical-tissue conditions, one classifier per condition, were converted into GSEA format (gmt, gene matrix transposed) and organized into two files according to their tissues of origin (brain or ovary). Each classifier composed of multiple gene features became a gene set. Gene features were represented by their corresponding gene symbols [30], if available on Agilent zebrafish annotations, or original probe identifications (IDs). The two gene set files were tested on both the original training dataset and a newly generated validation dataset. This new set of GEPs was generated from fish exposed concurrently with the other fish used previously for the training data. A total of 28 samples, 19 ovaries and nine brains, and their paired controls were profiled for their gene expression. Labeling and hybridization protocols employed for these new data were similar to those used for the training data; however, a newer version of Agilent zebrafish microarray (G2519F, design 019161) had to be used because the previous design 015064 was discontinued. The ovary samples included eight treated with flutamide and eight treated with prochloraz. And there were another eight brain samples from fish treated with prochloraz as well. These chemical-tissue conditions were selected because they had a large number of TF networks each generating a qualified classifier. Similar to the previous training data, half of the samples in each of these three treatment conditions were exposed for 48 hr and the other half for 96 hr. Each sample was also paired to its corresponding control. The remaining four samples were extra controls from these exposures included for microarray data preprocessing only. Outputs from Agilent Feature Extraction software were organized into three datasets, brain only, ovary only, brain and ovary combined, and processed as single channel intensities following a previously described procedure [18]. These three different ways of organizing the same outputs provided an opportunity to assess whether microarray data preprocessing would be impacted by sample size.

Given that the data for training and validation were produced from Agilent zebrafish microarrays of two different designs, their probe IDs had to be cross mapped (Additional file 3: Table S2). Several types of evidence were considered when cross-mapping the probe IDs between the designs: probe sequences, probe coordinates along a chromosome (019161_D_BED_20110527_modified.bed, 015064_D_BED_20110527_modified.bed made available by Agilent as of June 14, 2011; bed, browser extensible data), GenBank reference sequence (RefSeq; zebrafish.rna.fna as of June 20, 2011), Ensembl gene sequences (32312 unspliced including 5' UTR, exons, introns, 3' UTR) [31], and Ensembl cDNA sequence (Danio_rerio.Zv9.62.cdna.abinitio.fa, Danio_rerio.Zv9.62.cdna.all.fa as of June 14, 2011). Probes were mapped by common sequence targets on cDNA, RefSeq, and genes by BLASTN at a minimum E-value of E-10, or perfect match of probe sequences, or +/− 50 bps of start positions on the same chromosome. Number of probes matched between the designs varied with type of evidence (Additional file 3: Table S2). Overall, 24677 of 43603 probes from the design 019161 were matched to 17302 of 21495 probes from the design 015064.

A typical classification algorithm such as SVM relies on a training dataset and its derived computational model to make a prediction on an unknown test sample. It is difficult to evaluate the performance of a gene classifier on its own independent of the original training data. Two alternative approaches were adopted to address this issue, one statistical and the other by visual examination of heat maps. In GSEA [32], a classifier originating from a known chemical-tissue condition was validated on the newly produced GEPs. In the current study, qualified classifiers for various conditions were organized into gene sets by brain or ovary, and analyzed with either the original training data or the newly generated GEPs dedicated for validation. Each classifier contained gene features unique only to its own chemical-tissue condition and not shared with any other classifiers. The gene sets were paired to a microarray dataset with the same tissue type. GSEA was conducted using an R script wrapper and the release GSEA.1.0.R from the Broad Institute [18,33]. Based on the GSEA results, selected heat maps were generated for prochloraz gene classifiers from brain and ovary using their respective, tissue-specific training and validation datasets. To function effectively as a positive control, GSEA was also conducted for all the classifiers on the original training datasets of brain and ovary. For both GSEA and heat maps, gene features were cross mapped between the microarray design 015064 and 019161 based on the procedure and outputs described above, but at an elevated stringency of E-25 for BLAST-based evidence (Ensembl genes, cDNA, GenBank RefSeq) and +/− 20 bps for probe starting positions (Agilent bed files).

## Results

### Putative classifier identification and their distribution across chemicals, tissues, and networks

**Figure 2:**
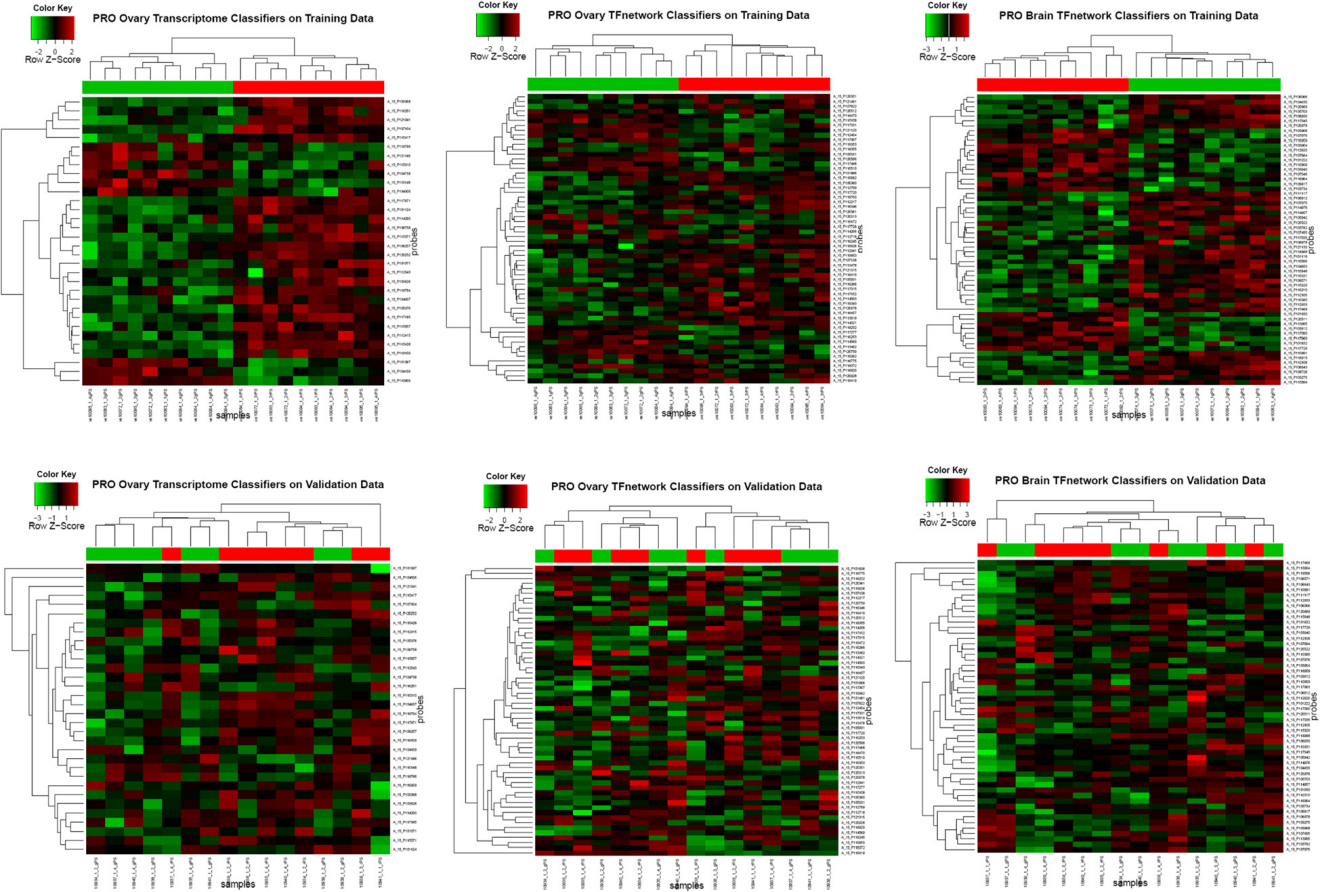
**A comparison of heat maps of selected gene classifiers.** Heat maps were generated for gene classifiers of prochloraz-ovary and prochloraz-brain based on the corresponding gene expression profiles of the same chemical-tissue conditions in the respective training and validation datasets. All datasets were prepared tissue-specifically. The classifiers were unique to each chemical-tissue condition, and cross mapped between the design 015064 and 019161 based on the exact match of their probe sequences. The red and green bars above each heat map indicate treated and control samples in a condition. Pairs of heat maps in each column compare a classifier of the same group of gene features between the training and validation data. Pro, prochloraz; TF network, transcription factor network.

In the current study, microarray data were organized and prepared according to individual tissue types (brain, ovary, and testis) each containing multiple chemicals (Table 1). Each condition may also contain data from multiple time points of exposure. Several brain conditions also had mixed male and female fish, with seemingly minimal impact on the discovery and performance of their classifiers. The purpose of pooling data in these conditions was to have increased sample size for their classifier search. A separate dataset was constructed by combining all three tissue types together along with additional liver samples for 17α-ethynyl estradiol, flutamide, ketoconazole, and 17β-trenbolone. Searches for gene classifiers were conducted for individual chemicals and their controls in these four datasets (brain, ovary, testis, all tissues combined) either across the entire zebrafish transcriptome or within each of the 517 individual networks (Tables 2, 3). Given the nature of binary searches, a classifier discovered in this study was intended only for distinguishing GEPs from fish exposed to a chemical or control within a particular tissue, and not for diagnosing between chemicals. While most of these 10,000 plus searches yielded putative gene classifiers, only a small subset of them was later qualified by visual evaluation of their heat maps (Figure 2). Unless otherwise explicitly stated, it is these qualified classifiers that are analyzed and discussed further. In addition, throughout this report, a classifier will always be discussed in the context of a chemical-tissue condition. For example, a classifier for prochloraz-ovary would refer to a number of gene features identified from the ovary microarray data of prochloraz-treated fish. If valid, it should diagnose GEPs of fish with the same exposure history.

**Table 2:** Chemical-tissue conditions found with (1) or without (0) a qualified gene classifier

**Chemical/tissue**	**All tissues**	**brain**	**ovary**	**testis**
17α-Ethynyl Estradiol	0	0	---	0
Fadrozole	0	0	0	*NA*
Fipronil	0	0	0	1
Flutamide	0	*NA*	1	0
Ketoconazole	0	0	1	---
Muscimol	0	1	---	*NA*
Prochloraz	0	0	1	1
17β-Trenbolone	0	---	0	*NA*
Trilostane	0	*NA*	---	0
Vinclozolin	0	*NA*	0	0

Searches were conducted transcriptome-wide using genetic algorithm-support vector machine followed by visual screening of heat maps. Each condition refers to the tissue sampled from fish treated with a particular chemical. “NA”, a condition not included in the study; “---“, insufficient sample size for classifier search.

**Table 3:** Summary statistics of qualified gene classifiers from network-specific searches

**Chemical/tissue**	**All tissues (495 search / condition)**^**a**^	**Brain (496 search / condition)**	**Ovary (495 search / condition)**	**Testis (497 search / condition)**	**Subtotal by chemical**
Prochloraz	&	177	68	76	321
Flutamide	&	*NA*	124	0	124
Fipronil	&	71	17	20	108
17α-ethynyl estradiol	0	41	---	50	91
Vinclozolin	0	*NA*	28	1	29
Ketoconazole	&	18	7	---	25
Muscimol	&	22	---	*NA*	22
Fadrozole	&	4	0	*NA*	4
17β-trenbolone	&	---	0	*NA*	
trilostane	0	*NA*	---	0	
Subtotal by tissue		333	243	147	

A total of 517 individual networks were analyzed by chemical-tissue conditions using genetic algorithm-K nearest neighbors. Within a condition, each TF network contributed one classifier composed of multiple gene features. “NA”, a condition not included in the study; “---“, insufficient sample size for classifier search; “0”, searched but no qualified classifier; “&”, no search conducted.

^a^Between 20 to 22 of the 517 networks did not contain sufficient number of genes to be searched due to variations in their availability in different datasets.

Given the strong impact of a tissue type on an overall transcriptional profile, it is not surprising that no classifiers were discovered for any chemicals using the expression data combined from multiple tissues (Table 2). When searches were conducted across the entire zebrafish transcriptome using only data from individual tissue types, classifiers were found for six conditions: muscimol-brain, flutamide-ovary, ketoconazole-ovary, prochloraz-ovary, fipronil-testis, and prochloraz-testis (Table 2). A typical classifier for these conditions had approximately 50 gene features. Excluding those features shared among multiple conditions, a classifier for each of the six conditions had from 20 to 30 unique gene features.

In addition to searches across the entire zebrafish transcriptome, similar analyses were also conducted by TF networks. The search space for these was limited to the 517 individual networks, including a group of genes compiled for HPG axis and another group made up exclusively of hub TFs (master regulators), the networks of which had been previously linked to various chemical-tissue conditions (Table 3) [18]. While preliminary classifiers were found from the HPG axis and the master regulators respectively for all 19 possible chemical-tissue conditions, only one classifier (flutamide-ovary) from the master regulators later qualified based on its heat map. Among the remaining 515 TF networks searched, a number of qualified classifiers were found for each of 15 chemical-tissue conditions (Table 3) from a total of 255 unique networks, with one per TF network per condition. At maximum, gene features in a given TF network could be selected into classifiers for 10 different conditions, with a median of two (data not shown). The top contributing TF networks in this regard (as denoted by their hub TFs) included XBP1 (X-box binding protein), HIF1AB (hypoxia inducible factor), YBX1 (Y-box binding protein), SMAD2 (MAD homolog), and YY1B (YY1 transcription factor). No preliminary classifiers from fadrozole-ovary, 17β-trenbolone-ovary, flutamide-testis, and trilostane-testis were qualified (Table 3). Brain tissue had the greatest number of TF networks (333) each yielding a qualified classifier, followed by ovary (243) and testis (147). By individual chemicals, prochloraz had the greatest number of TF networks (321) each generating a classifier, followed by flutamide (124), fipronil (108), and 17α-ethynyl estradiol (91). A similar pattern was also observed in the distribution of number of gene features in a classifier (Table 4). Classifiers from brain had the most gene features (1894), followed by ovary (1299) and testis (643). Chemical-wise, classifiers for prochloraz had the greatest number of gene features (1848), followed by flutamide (814), and 17α-ethynyl estradiol (473) (Table 4). A majority of these gene features overlapped among multiple classifiers.

**Table 4:** Number of gene features in qualified classifiers for various chemical-tissue conditions

**Chemical**	**No. unique gene features (total)**			
	**Brain**	**Ovary**	**Testis**	**Subtotal by chemical**
Prochloraz	1236 (3391)	277 (1437)	335 (1550)	1848 (6378)
Flutamide	*NA*	814 (2491)	0	814 (2491)
17α-ethynyl estradiol	221 (897)	---	252 (737)	473 (1634)
Fipronil	261 (1370)	47 (338)	53 (353)	361 (2061)
Vinclozolin	*NA*	131 (662)	3 (6)	134 (668)
Ketoconazole	84 (519)	30 (185)	---	114 (704)
Fadrozole	14 (115)	0	*NA*	14 (115)
Muscimol	78 (656)	---	*NA*	78 (656)
17β-trenbolone	---	0	*NA*	
Trilostane	*NA*	---	0	
Subtotal by tissue	1894	1299	643	

Based on Agilent probe IDs, each total count includes those gene features overlapping among multiple classifiers. While search was network-specific, gene features were summarized across multiple networks in a classifier for a given condition where available. A total of 7522 features were selected into classifiers for the 15 conditions listed. “NA”, not included in the study; “---“, insufficient sample size for classifier search; “0”, searched but no qualified classifier.

### Testing/validation of selected classifiers

After their discovery from the original training dataset and passing visual screening of heat maps, classifiers were still considered as candidates until further validation on an independently generated microarray dataset. Using GSEA, a classifier defined by its original chemical-tissue condition, for example, prochloraz-ovary, was tested to see if it could correctly identify the exposure in an independent organism with the same exposure history. A total of six classifiers from the transcriptome-wide search and 14 classifiers from the network-specific search each had enough gene features unique only to a particular condition to be a useful gene set. To prove its utility as a diagnostic tool, GSEA of classifiers was also conducted on the original training data alongside with the new validation dataset. In theory, GSEA should link a classifier to the GEPs of the training data sharing a common chemical-tissue condition.

The results of testing classifiers were inconsistent. Discrepancies existed not only between the original training data and validation dataset, but also within each of them. As expected for the training data, nine out of the 11 gene classifiers identified the chemical exposures of their original GEPs as top candidates based on statistically significant enrichment of gene sets on ranked gene lists (Table 5). The classifier for flutamide-ovary also identified its target GEP of the same condition, but only as the third choice. However, in the majority of cases, several classifiers representing different chemical-tissue conditions would also identify the same GEP. For example, classifiers for prochloraz-ovary, ketoconazole-ovary, and flutamide-ovary all diagnosed the GEP of prochloraz-ovary. The GEP of fadrozole-brain, however, failed for all classifiers tested, including surprisingly its own.

**Table 5:** Gene set enrichment analysis (GSEA) of classifiers on their original gene expression profiles (GEPs) for training

**Chemical**	**Brain GEP (F-ratio)**	**Brain classifiers enriched on brain GEP (FDR)**	**Ovary GEP (F-ratio)**	**Ovary classifiers enriched on ovary GEP (FDR)**
Fadrozole (FAD)	**FAD-brain (3.41)**	No enrichment	FAD-ovary (2.01)^a^	PRO-ovary (0.005)
				KET-ovary (0.006)
				FLU-ovary (0.01)
17α-ethynyl estradiol (EE2)	**EE2-brain (5.96)**	EE2-brain (0.028)	EE2-ovary (22.4)^b^	KET-ovary (0.002)
				PRO-ovary (0.004)
				FLU-ovary (0.023)
Muscimol (MUS)	**MUS-brain (6.42)**	MUS-brain (0.0)	MUS-ovary (42.62)^b^	FIP-ovary (0.027)
				KET-ovary (0.035)
				PRO-ovary (0.052)
				FLU-ovary (0.077)
Fipronil (FIP)	**FIP-brain (6.78)**	FIP-brain (0.002); MUS-brain (0.012)	**FIP-ovary (10.23)**	FIP-ovary (0.006);
				PRO-ovary (0.0)
				KET-ovary (0.0)
Flutamide (FLU)	*NA*		**FLU-ovary (67.23)**	KET-ovary (0.002)
				PRO-ovary (0.006)
				FLU-ovary (0.007)
Ketoconazole (KET)	**KET-brain (9.17)**	KET-brain (0.006); MUS-brain (0.008)	**KET-ovary (4.85)**	KET-ovary (0.0)
				PRO-ovary (0.008)
				FLU-ovary (0.18)
Prochloraz (PRO)	**PRO-brain (19.42)**	PRO-brain (0.0)	**PRO-ovary (14.48)**	PRO-ovary (0.0)
				KET-ovary (0.009)
				FLU-ovary (0.008)
17β-trenbolone (TRE)	TRE-brain (22.94)^b^	No enrichment	TRE-ovary (3.28)^a^	KET-ovary (0.1)
				PRO-ovary (0.234);
				FIP-ovary (0.236)
Trilostane (TRI)	*NA*		TRI-ovary (11.16)^b^	PRO-ovary (0.121)
Vinclozolin (VIN)	*NA*		**VIN-ovary (6.09)**	VIN-ovary (0.024);
				KET-ovary (0.0)
				PRO-ovary (0.107)
				FLU-ovary (0.106)
	F-ratio mean = 10.59		F-ratio mean = 18.44	

Each classifier was formatted into a gene set, and multiple gene sets were grouped by their tissue of origin. GSEA was conducted by individual chemical-tissue conditions, at a threshold of FDR ≤ 0.25. Classifiers enriched on the top or bottom of a ranked gene list are separated by a semicolon. If two classifiers of the same condition are both significant, only the most significant one is listed. A GEP in bold indicates the availability of its corresponding classifier as a gene set. FDR, false discovery rate; F-ratio, maximum Fisher’s discriminant ratio; “NA“, a condition not included in the study.

^a^Searched but no qualified classifier found for the condition. Its GSEA was conducted using classifiers for other unrelated chemical-tissues.

^b^No classifier search was conducted due to insufficient sample size for the condition. Its GSEA was conducted using classifiers for other unrelated chemical-tissues.

While testing classifiers on their original training data proved the utility of GSEA as a diagnostic tool, a classifier has to be evaluated on data completely independent from its discovery. The results were also mixed when selected classifiers from brain and ovary were tested separately on the validation dataset (Table 6). Since the validation datasets prepared by ovary, brain, and their combination all gave similar results, only GSEA results based on the combined brain and ovary data are presented. Despite potential issues resulting from cross-mapping probes between the two microarray designs or slight differences in sample preparation, the GEP of flutamide-ovary was diagnosed by its corresponding classifier, and by classifiers for vinclozolin-ovary, ketoconazole-ovary, and prochloraz-ovary as well. Similarly, the GEP of prochloraz-ovary was also identified by its classifier and that of ketoconazole. In contrast, none of the classifiers was able to identify the GEP of prochloraz-brain. When the classifiers for prochloraz-brain and prochloraz-ovary were evaluated visually by their heat maps on the corresponding GEPs of the same chemical-tissue conditions from the original training and validation datasets, the complete separation of treated and control samples observed in the training datasets was not evident in the validation data (Figure 2).

**Table 6:** Gene set enrichment analysis (GSEA) of classifiers on their validation data

**chemical**	**Brain GEP (F-ratio)**	**Brain classifiers enriched on brain GEP (FDR)**	**Ovary GEP (F-ratio)**	**Ovary classifiers enriched on ovary GEP (FDR)**
Flutamide (FLU)	*NA*	*NA*	FLU-ovary (13.76)	VIN-ovary (0.174)
				FLU-ovary(0.21)^a^;
				KET-ovary (0.039)
				PRO-ovary (0.123)
Prochloraz (PRO)	PRO-brain (24.75)	No enrichment	PRO-ovary (15.2)	PRO-ovary (0.067)
				KET-ovary (0.163)

Although the gene expression profiles (GEPs) were preprocessed as a whole by combining data from both brain and ovary, each GSEA was conducted by individual chemical-tissue conditions at a threshold of FDR ≤ 0.25. Classifiers enriched on the top or bottom of a ranked gene list are separated by a semicolon. If two classifiers for the same condition were both significant, only the most significant one is listed. Mapping of the probe IDs between the Agilent design 15064 and 19161 was based on identical probe sequences only. FDR, false discovery rate; F-ratio, maximum Fisher’s discriminant ratio. “NA”, a condition not included in the study.

^a^When the GEPs were preprocessed with data from ovary only, the FDR for flutamide-ovary was 0.53.

### Data complexity of chemical-tissue conditions

Given the impact of data complexity on the performance of gene classifiers, it is potentially informative to bring this measure into the discussion. A smaller F-ratio reflects less separation of the population mean responses of treated and controls in a given condition relative to their variances, thus a greater complexity.

Within the training data, fadrozole-brain had the smallest F-ratio (3.41), followed in ascending order by (in the brain) 17α-ethynyl estradiol, muscimol, fipronil, ketoconazole, prochloraz, and 17β-trenbolone (22.94; Table 5). In ovary, fadrozole again had the smallest F-ratio (2.01), followed by 17β-trenbolone, ketoconazole, vinclozolin, fipronil, trilostane, prochloraz, 17α-ethynyl estradiol, muscimol, and flutamide (67.23). Between the two tissues, the relative ranking order of F-ratios remained similar for some chemicals but changed with others, notably 17α-ethynyl estradiol, muscimol, and 17β-trenbolone. There was also a several-fold difference between the largest F-ratios in ovary versus the brain, of flutamide-ovary and 17β-trenbolone-brain respectively. On average, the GEPs of ovary had a greater F-ratio (18.44) than those of brain (10.59).

The F-ratios of several chemical-tissue conditions were not consistent between the training and validation datasets. In the training set, several selected conditions with F-ratios from low to high were prochloraz-ovary (14.48), prochloraz-brain (19.42), and flutamide-ovary (67.23). In the validation data, the sequence changed to flutamide-ovary (13.76), prochloraz-ovary (15.2), and prochloraz-brain (24.75; Table 6). Also notable was an observation that those conditions with smaller F-ratios (fadrozole-brain, fadrozole-ovary, 17β-trenbolone-ovary) in the training data tended to yield fewer classifiers while those with greater F-ratios (flutamide-ovary, prochloraz-brain, prochloraz-ovary) tended to yield more (Table 3).

## Discussion

Perhaps the most critical component of any gene classifier discovery effort is independent validation. Especially in the field of ecotoxicology, there is a current prevalence of candidates but few field-ready classifiers for exposure and risk assessment. With a limited number of biological replicates in most microarray studies due to cost considerations, it is not clear how well these transcriptomics-based gene classifiers will perform in validations. Rather than directly pursuing more complicated multi-class classifiers in our current study, a choice was made to discover simpler binary classifiers for two reasons. One was to avoid potential computational issues arising from the complex biological interactions of EDCs all targeting the various components of the HPG axis. Second, a multi-class problem can be decomposed into a number of binary classifications problems [34], and initial binary classifiers successfully identified could later be combined computationally to tackle a multi-class classification problem [35]. While the microarray training dataset used in this study was not designed specifically for gene classifier discovery, it does represent typical microarray data obtained in ecotoxicology experiments, both in terms of the extent of biological sample replication, and the relevance of EDCs to chemicals of concern in fish and wildlife.

In order to make predictions on samples of interest of their chemical exposure history, a typical classification algorithm requires the availability of training data or its representation as a computational model derived beforehand. An approach like GSEA offers a flexible alternative. There are two possible scenarios for interpretation when GSEA determines that a classifier as assayed on samples of interest is enriched on the rank-ordered gene lists generated from reference GEPs associated with a known chemical. For an established classifier on samples of unknown, the chemical exposure history of these samples would be diagnosed by the chemical condition associated with the reference GEPs. For a candidate classifier on known samples, on the other hand, a connection of corresponding chemical conditions between these samples and reference GEPs would effectively validate the classifier. In a typical study of biomarkers, the same algorithm is used in their discovery and validation. In this research, however, independent algorithms were used in the two stages. The successful linkage, in 10 out of a total of 11 cases, of treatment conditions of classifiers to those of their respective GEPs in the training data demonstrated not only the strength of GA-SVM/GA-KNN for discovery, but also the utility of GSEA for validation.

The inconsistent predictive performance of several classifiers on both the original training data and later-generated validation data could be attributed in part to the data complexity of their respective chemical tissue conditions unresolved by computational modeling at their existing sample size. This complexity reflects the differential biological impact of an EDC on an individual fish relative to its overall population. In other words, with all other factors equal, the performance of a classifier is probably a function of both the (mechanisms of) action of the EDC and the population variation in organisms’ response to exposure of these chemicals, both genetic and environmental. Accordingly, the sample size required for classifier discovery would likely vary among different chemical-tissue conditions. When biological replication is inadequate for a given level of data complexity, model overfitting could result in an incorporation of both signal and noise, leading to unsuccessful predictions later [36,37].

As one of a variety of measures proposed to capture the data complexity of GEPs, F-ratio statistic varies in its degree of correlation with the performance of classifiers[11,12,14], probably because of a wide range of linear separability present among microarray datasets [13]. Many complexity measures including F-ratio are thought to be especially effective in characterizing linearly separable data [10]. In the current study, a similar inconsistent correlation was also observed between F-ratio and classifier performance. In conditions like fadrozole-brain, prochloraz-brain, and prochloraz-ovary in the training data, F-ratio reflected the performance of classifiers (Table 5). In other conditions such as prochloraz-brain across the training and validation data, this correlation broke down (Table 6). And the F-ratio of the same condition, for example flutamide-ovary, also varied widely between the training and validation data in spite of the fact that fish samples involved were co-exposed in the same batch. Similar challenges of data complexity impacting classifier performance were also observed in cancer classification data [15]. Thus, a better characterization of microarray data complexity probably requires an exploration of a full suite of statistical measures and depends highly on chemical-tissue conditions.

The discrepancy between GSEA validation results and the corresponding heat maps is difficult to interpret. The heat maps of prochloraz classifiers achieved a perfect separation of treated and control samples from the training data as expected. Given the partial success of these same classifiers on the validation data by GSEA, it was expected that this would also be reflected in the heat maps. While model overfitting cannot be completely ruled out here, it is likely that such a strict bifurcation of samples in heat maps via two-way clustering of both gene classifiers and samples is simply a more stringent criterion than FDR 0.25, a commonly accepted threshold for GSEA. Also notable is that, while GA-SVM/GA-KNN generated classifiers from the entire transcriptome for most of the conditions or from most individual TF networks for a given condition, a majority of these classifiers were later disqualified during visual examination of their heat maps under a strict requirement of bifurcation of treated and control samples. Some of these disqualified classifiers could be false negatives.

While the chemicals included in this study all target the HPG axis and preliminary classifiers were indeed found from this compiled group of genes for all 19 conditions, none of these classifiers was later qualified by their heat maps. A similar pattern was also observed in master regulators, a group of hub TFs anchoring gene regulatory networks previously found to be associated with various chemical-tissue conditions for this same set of EDCs [18]. In contrast, a previous study of master regulators suggested that they tended to be stable and reliable classifiers [9]. In addition, almost half of the 515 networks generated a qualified classifier for at least one condition. There are several possible explanations for these observations. First, strict separation of treated and control samples in a heat map might be a criterion too stringent for a qualifying classifier. Second, the target genes in the HPG axis operate in a variety of tissue types [38] and many of them respond to multiple chemicals. Within a given tissue, these genes may not be involved in endocrine regulatory functions. And lastly, with regard to the master regulators, hub TFs may just be classifiers more suitable for problems with better-defined biological endpoints, as in the case of cancer morphology, than for chemicals with diverse treatment effects [15].

With binary classifiers found for six chemical-tissue conditions by the transcriptome-wide searches, and 15 conditions by the network-specific searches, each containing many gene features, their distribution statistics across chemicals, tissues, and TF networks could offer some biologically important insights into the MOAs of EDCs. Assuming a positive connection between biological impact and the number of such classifiers and gene features involved, the 10 EDCs under study had the greatest effects on brain tissue, then ovary and testis. Chemical-wise, prochloraz would rank as the most active chemical (in terms of networks affected), followed by flutamide, fipronil, 17α-ethynyl estradiol, vinclozolin, ketoconazole, muscimol, fadrozole, 17β-trenbolone, and trilostane. Another interesting perspective came from the top TF networks which were shown to generate classifiers for a number of chemical-tissue conditions. Some of these networks, as denoted by their hub TFs, included XBP1, HIF1AB, YBX1, SMAD2, and YY1B. The cellular functions behind these TFs include stress response (XBP1) [39], regulation of oxygen homeostasis (HIF1AB) [40], regulation of transcription and/or translation (YBX1, YY1B) [41,42], and signal transduction (SMAD2) [43], suggesting far-reaching biological effects of the test chemicals on zebrafish beyond the HPG axis.

With a current knowledge of molecular pathways associated with different types of chemical stressors often lacking, an attempt was made in this study to develop more mechanistically-based gene classifiers by conducting TF network-specific searches. The idea behind this approach was to first construct transcriptome-wide TF networks based on the GEPs from fish samples treated with multiple EDCs, and then to identify a subset of these networks significantly impacted by individual endocrine-active chemicals. Classifiers later identified specific to these individual networks would in effect be tied to EDCs through common TF networks, which would add a mechanistic perspective to these classifiers. In comparison, a classifier from the transcriptome-wide search was more likely to be a biologically random assemblage of gene features. Based on hundreds of individual TF networks previously constructed and linked to various EDCs by GSEA [18], the network-specific search generated a substantial number of classifiers for multiple conditions in this study despite greatly reduced search space. This approach also yielded classifiers for more chemical-tissue conditions than the transcriptome-wide search. Upon confirmation, these classifiers could provide an explicit linkage among a chemical stressor, its underlying regulatory TF networks, and apical (whole animal) responses.

A common pattern emerging from both training and validation datasets is that multiple classifiers sharing no common gene features could predict the same target GEP. Assuming a subset of these classifiers is valid, such an overlapping prediction reflects complex interactions between the test chemicals and biological pathways within the tissues that comprise the HPG axis [17,18], some of which are expected according to the current biological understanding of these chemicals. For example, among the 10 EDCs, both vinclozolin and flutamide are androgen receptor antagonists in multiple androgen/estrogen-responsive tissues while ketoconazole, fadrozole, and prochloraz are overlapping inhibitors of cytochrome P450 11A, 17, and 19 respectively in the gonad. In both the training and validation data, the classifiers for these conditions often simultaneously diagnosed the same GEP, as is the case between prochloraz and ketoconazole. Perhaps more significant is when this relationship of multiple classifiers all predicting a single GEP occurred with seemingly unrelated EDCs, such as prochloraz and fipronil, the latter a GABA (gamma-amino butyric acid) receptor antagonist. Such a many-to-many relationship among classifiers and GEPs from different conditions would be informative with regard to the underlying pathways affected by these chemicals and their possible grouping into a smaller number of classes, thus reducing the need to develop classifiers for individual chemical-conditions.

GSEA proved to be a flexible tool in ecotoxicological application of gene classifiers without relying on their original training data. Future improvement could be made by adopting Cmap, a conceptually similar but more refined approach developed for human biomedical studies [8]. Cmap connects small molecule drugs to one another or a small molecule drug to a human disease state by mapping a group of gene signatures to GEPs previously assembled as a large reference collection of ranked gene lists. Each GEP is generated from samples treated with a chemical of interest. With its demonstrated flexibility and effectiveness [44], the Cmap algorithm could be an excellent analytical tool for ecotoxicological applications. The main barrier to its application is the need to generate and assemble a large collection of GEPs linked to a wide variety of chemical compounds of environmental significance. Conceivably, Cmap could be used in conjunction with preexisting gene classifiers to make predictions on field samples with unknown exposures, validate candidate gene classifiers using pre-established reference GEPs, categorize environmental stressors into a more manageable number of chemical classes based on pathways affected, and link chemical stressors to their apical biological endpoints.

## Conclusions

In summary, binary gene classifiers for a number of chemical-tissue conditions were identified based on the GEPs of fish samples previously exposed to these chemicals. Consistent with prior reports, tissue type was found to have a major impact on a transcriptome such that data containing multiple tissues yielded no qualified classifiers. The distribution characteristics of classifiers across tissues, chemicals, and TF networks suggested a differential biological impact among the chemicals on zebrafish transcriptome involving some basic cellular functions. An independent validation of classifiers for prochloraz-ovary, flutamide-ovary, and prochloraz-brain on additional GEPs was partially successful. Data complexity measured by F-ratios varied among individual chemicals and between tissue types, suggesting sample size required for classifier discovery is likely different for various chemical-tissue conditions. The varying data complexity was probably also partially responsible for inconsistent performance of classifiers observed in the current study. In the future, a preliminary survey of transcriptomic impact by various stressors would likely be informative with regard to their data complexity and appropriate sample size for gene classifier discovery. An adoption of Cmap-based approach for ecotoxicological applications of gene classifiers may also provide a more effective and flexible tool for exposure and risk assessment.

## Endnotes

^a^While terms such as gene classifier, gene signature, molecular indicator, biomarker and their respective plural forms are often used interchangeably in literature, the term gene classifier is used throughout this paper. In its singular form, a gene classifier is defined here as a group of microarray gene features (signatures, probes) capable of distinguishing samples in a single chemical-tissue condition between the treated and control. Gene classifiers, on the other hand, refer to multiple sets of gene features each diagnostic of a different chemical-tissue condition.

## Abbreviations

Cmap: Connectivity map; BED: Browser extensible data; EDC: Endocrine-disrupting chemicals; FDR: False discovery rate; F-ratio: Fisher’s discriminant ratio; GA-SVM: Genetic algorithm-support vector machine; GA-KNN: Genetic algorithm-K nearest neighbors; GEP: Gene expression profile; GSEA: Gene set enrichment analysis; HPG: Hypothalamic-pituitary-gonadal; MOAs: Mechanisms of action; TF: Transcription factor.

## Competing interests

The authors declare that there are no competing interests.

## Authors’ contributions

RLW conducted all the bioinformatic analyses and prepared the manuscript. DB, AB, and RF contributed to microarray work. DV and GA conducted fish exposure to various chemicals. JL was involved in the project planning and execution. All coauthors contributed to the manuscript revisions and approved the final version for submission.

## Supplementary Material

Additional file 1 **Figure S1.** An illustration of GALGO algorithm as adapted from GALGO tutorial (Figure 10 and 22, version February 2006; http://biptemp.bham.ac.uk/vivo/galgo/Tutorial.pdf). Click here for file

Additional file 2 **Table S1.** Configurations for GA-SVM and GA-KNN search. Click here for file

Additional file 3 **Table S2.** Cross mapping probe IDs between the design 015064 and 019161 by five types of evidence: Agilent BED (browser extensible data) files, Zv9 cDNA and genes from Ensemble, reference sequences (RefSeq) from GenBank, and Agilent probe sequences. Click here for file
