# Supplementary material for: Discovery and validation of gene classifiers for endocrine-disrupting chemicals in zebrafish (danio rerio)
Source: BMC Genomics. 2012 Aug 1;13:358. doi: 10.1186/1471-2164-13-358 (PMC3469349; doi:10.1186/1471-2164-13-358)
Supplement: Additional file 1 — Figure S1. An illustration of GALGO algorithm as adapted from GALGO tutorial (Figure 10 and 22, version February 2006; http://biptemp.bham.ac.uk/vivo/galgo/Tutorial.pdf). [file 1471-2164-13-358-S1.pptx]

## Slide 1
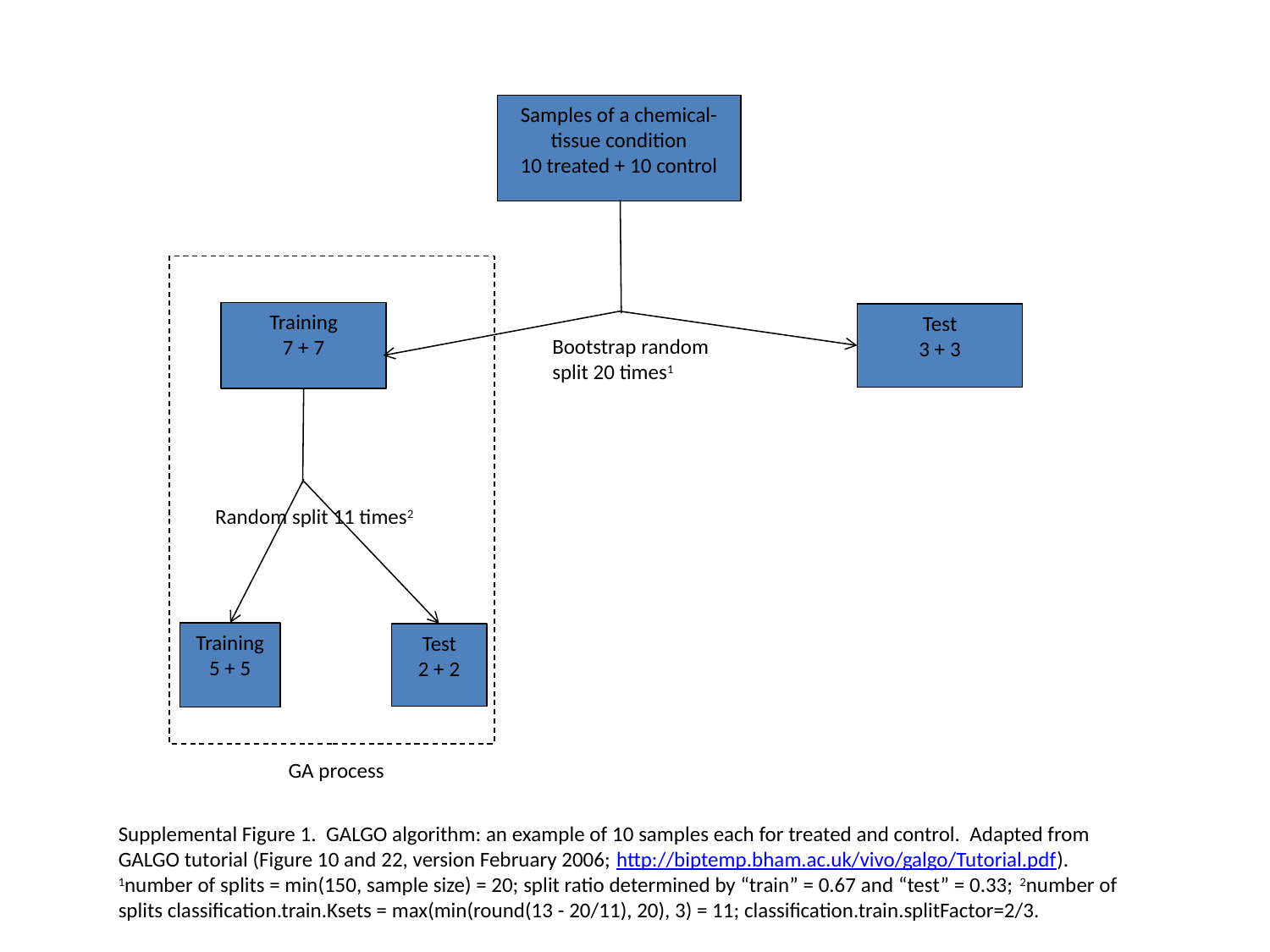

Samples of a chemical-tissue condition
10 treated + 10 control
Training
7 + 7
Test
3 + 3
Bootstrap random split 20 times1
Random split 11 times2
Training
5 + 5
Test
2 + 2
GA process
Supplemental Figure 1. GALGO algorithm: an example of 10 samples each for treated and control. Adapted from GALGO tutorial (Figure 10 and 22, version February 2006; http://biptemp.bham.ac.uk/vivo/galgo/Tutorial.pdf). 1number of splits = min(150, sample size) = 20; split ratio determined by “train” = 0.67 and “test” = 0.33; 2number of splits classification.train.Ksets = max(min(round(13 - 20/11), 20), 3) = 11; classification.train.splitFactor=2/3.
