# Supplementary material for: Discovery and validation of gene classifiers for endocrine-disrupting chemicals in zebrafish (danio rerio)
Source: BMC Genomics. 2012 Aug 1;13:358. doi: 10.1186/1471-2164-13-358 (PMC3469349; doi:10.1186/1471-2164-13-358)
Supplement: Additional file 2 — Table S1. Configurations for GA-SVM and GA-KNN search. [file 1471-2164-13-358-S2.doc]

Supplemental Table 1. Configurations for GA-SVM and GA-KNN search.

| **parameters** | **GA-SVM)** | **GA-KNN** |
| --- | --- | --- |
| Train | 0.67 | 0.67 |
| Test | 0.33 | 0.33 |
| Classification.method | Svm | Knn |
| Classification.test.error | c(0,1) | c(0,1) |
| Classification.train.error | splits | Splits |
| Classification.train.Ksets | -1 | -1 |
| Classification.train.splitFactor | 2/3 | 2/3 |
| Classification.rutines | R | --- |
| Classification.userFitnessFunc | NULL | NULL |
| Scale | TRUE | TRUE |
| Svm.kernel | linear | --- |
| Svm.type | C-classification | --- |
| Svm.nu | 0.5 | --- |
| Svm.degree | 4 | --- |
| Svm.cost | Dataset specific grid search | --- |
| Knn.k | --- | 5 |
| Knn.l | --- | 3 |
| Knn.distance | --- | euclidean |
| geneFunc | runifInt | runifInt |
| chromosomeSize | 20 | 20 |
| populationSize | -1 | -1 |
| niches | 2 | 2 |
| Worlds | 1 | 1 |
| Immigration | c(rep(0,18),0.5,1) | c(rep(0,18),0.5,1) |
| offspringScaleFactor | 1 | 1 |
| offspringMeanFactor | 0.85 | 0.85 |
| offspringPowerFactor | 2 | 2 |
| Elitism | c(rep(1,9),0.5) | c(rep(1,9),0.5) |
| goalFitness | 0.9 | 0.9 |
| galgoVerbose | 20 | 20 |
| maxGenerations | 200 | 200 |
| minGenerations | 10 | 10 |
| galgoUserData | NULL | NULL |
| maxBigBangs | 1000 | 500 |
| maxSolutions | 1000 | 500 |
| saveVariable | OutputID | OutputID |
| saveFrequency | 30 | 30 |
| callBackFuncBB | NULL | NULL |
