# Supplementary material for: Discovery and validation of gene classifiers for endocrine-disrupting chemicals in zebrafish (danio rerio)
Source: BMC Genomics. 2012 Aug 1;13:358. doi: 10.1186/1471-2164-13-358 (PMC3469349; doi:10.1186/1471-2164-13-358)
Supplement: Additional file 3 — Table S2. Cross mapping probe IDs between the design 015064 and 019161 by five types of evidence: Agilent BED (browser extensible data) files, Zv9 cDNA and genes from Ensemble, reference sequences (RefSeq) from GenBank, and Agilent probe sequences. [file 1471-2164-13-358-S3.docx]

Supplemental Table 2. Cross mapping probe IDs between the design 015064 and 019161 by five types of evidence: Agilent BED (browser extensible data) files, Zv9 cDNA and genes from Ensemble, reference sequences (RefSeq) from GenBank, and Agilent probe sequences. For Agilent BED files, a maximum gap allowance of ≤ 50 bps was set between the start positions of any two probes from the two designs in order to be considered identical. The probes from the two designs were also linked by identical probe sequences, or sharing common hits on Ensembl Zv9 cDNA, genes, and GenBank reference sequences by BLASTN with a minimum E value of 1e-10. The total number of unique probes mapped between the design 019161 and 015064 are 24677 out of 43603, and 17302 out of 21495 respectively.

| No. match 019161/015064 | 015064 BED file | 015064 Zv9 cDNA | 015064 Zv9 gene | 015064 RefSeq | 015064 probe sequence |
| --- | --- | --- | --- | --- | --- |
| 019161 BED file | 11974/12134 | --- |  | --- | --- |
| 019161 Zv9 cDNA | --- | 17502/14336 |  | --- | --- |
| 019161 Zv9 gene | --- | --- | 21433/14822 | --- | --- |
| 019161 RefSeq | --- | --- |  | 16403/13764 | --- |
| 019161 probe sequence | --- | --- |  | --- | 11227/11226 |
